# Oropharyngeal *Staphylococcus aureus* is linked to higher mortality in long-term aged care residents

**DOI:** 10.1093/ageing/afaf042

**Published:** 2025-03-03

**Authors:** Sophie J Miller, Frank Zhang, Steven Taylor, Richard Woodman, Andrew P Shoubridge, Lito E Papanicolas, Geraint B Rogers

**Affiliations:** Lifelong Health, South Australian Health and Medical Research Institute, Adelaide, South Australia, Australia; College of Medicine and Public Health, Flinders University, Bedford Park, South Australia, Australia; Microbiology and Infectious Diseases, SA Pathology, Adelaide, South Australia, Australia; Lifelong Health, South Australian Health and Medical Research Institute, Adelaide, South Australia, Australia; College of Medicine and Public Health, Flinders University, Bedford Park, South Australia, Australia; College of Medicine and Public Health, Flinders University, Bedford Park, South Australia, Australia; Lifelong Health, South Australian Health and Medical Research Institute, Adelaide, South Australia, Australia; College of Medicine and Public Health, Flinders University, Bedford Park, South Australia, Australia; Lifelong Health, South Australian Health and Medical Research Institute, Adelaide, South Australia, Australia; College of Medicine and Public Health, Flinders University, Bedford Park, South Australia, Australia; Microbiology and Infectious Diseases, SA Pathology, Adelaide, South Australia, Australia; Lifelong Health, South Australian Health and Medical Research Institute, Adelaide, South Australia, Australia; College of Medicine and Public Health, Flinders University, Bedford Park, South Australia, Australia

**Keywords:** microbiome, older people, nursing homes, methicillin-resistant *Staphylococcus aureus* (MRSA), methicillin-susceptible *S. aureus* (MSSA)

## Abstract

**Background:**

Biological ageing, healthcare interactions, and pharmaceutical and environmental exposures in later life alter the characteristics of the oropharyngeal (OP) microbiome. These changes, including an increased susceptibility to colonisation by pathobiont species, have been linked with diverse health outcomes.

**Objectives:**

To investigate the relationship between OP microbiome characteristics and all-cause mortality in long-term aged care residents.

**Methods:**

OP swabs were collected from 190 residents of five aged care facilities in South Australia. Microbiota composition was assessed by shotgun metagenomics and related to health outcomes during a 12-month follow-up period. OP carriage of *Staphylococcus aureus* and methicillin resistance was confirmed by qPCR.

**Results:**

OP carriage of *S. aureus* was identified in 13 (6.8%) residents. Detection of *S. aureus* was significantly associated with an increased risk of mortality (adjusted HR [95% CI]: 9.7 [3.8–24.9], *P* < .0001), compared with non-carriers, independent of methicillin resistance. *Staphylococcus aureus* carriage demonstrated a stronger association with mortality risk than the total number of comorbidities at the univariate level (*S. aureus* HR [95% CI]: 7.2 [3.4–15.5], *P* < .0001 vs. comorbidity count HR [95% CI]: 1.1 [1.0–1.3], *P* = .03), and remained significant after multivariable adjustment. *Staphylococcus aureus* detection was significantly associated with total number of comorbidities (adjusted OR [95% CI]: 1.4 [1.0–2.0], *P* = .04).

**Conclusion:**

OP *S. aureus* carriage predicts all-cause mortality in long-term aged care. We speculate that *S. aureus* carriage represents a marker of general health, including prior healthcare exposures. OP *S. aureus* carriage could contribute to estimations of general health in older individuals and thereby inform care strategies.

## Key Points

- Ageing and healthcare exposures alter the oropharyngeal (OP) microbiome, potentially influencing health outcomes.
- OP *Staphylococcus aureus* carriage is significantly associated with higher all-cause mortality in long-term aged care residents.
- The association between *S. aureus* carriage and mortality was independent of methicillin resistance status.
- Number of comorbidities was independently associated with *S. aureus* detection.
- OP *S. aureus* carriage may indicate poor health outcomes and inform care strategies in long-term residential aged care.

## Introduction

The oropharyngeal (OP) microbiome is the community of microorganisms that reside naturally within the oropharynx. These microbial communities exert an important influence on health through their interaction with the host immunity [1] and their suppression of opportunistic pathogens [2]. In later life, ageing-related changes in airway physiology [3, 4], immune function [5], pharmaceutical exposures [6, 7] and health services interactions [7] alter OP microbiome characteristics, contributing to adverse health outcomes [8, 9].

One consequence of ageing-associated OP microbiome changes is an increased susceptibility to asymptomatic colonisation by pathobionts species [10]. For example, asymptomatic upper airway colonisation by *Staphylococcus aureus* or *Streptococcus pneumoniae* is linked to poor clinical outcomes, and serves as a potential marker of declining health status and disease vulnerability [11, 12].

Advances in culture-independent microbiology enable identification of microbiome features that are associated with important health outcomes. Alignment of these features with health exposure data, including health services utilisation, comorbid conditions and pharmaceutical exposures, allows microbial markers or mediators of health outcomes to be identified. Our analysis focused specifically on the relationship between OP microbiome characteristics and all-cause mortality among long-term residential aged care residents.

## Methods

OP swabs were collected as part of the GRACE study, a cross-sectional investigation involving five long-term aged care facilities in metropolitan South Australia between March 2019 and March 2020. Health outcomes, including mortality, were assessed during a 12-month follow-up period [13]. No confirmed cases of COVID-19 were reported among aged care residents in South Australia during this time [14].

Sample collection and processing are detailed in Appendix 1. Briefly, microbiota composition was determined by shotgun metagenomic sequencing (Illumina Novaseq6000 platform with 150-bp paired end reads). Taxonomic composition data was determined using MetaPhlAn (v3.0). *Staphylococcus aureus* detection was confirmed by TaqMan qPCR targeting the single-copy *nuc* gene [15]. Methicillin resistance in *nuc*-positive samples was determined using SYBER Green qPCR *mecA* assay [16].

Individual associations between OP bacterial species and mortality were investigated using univariate Cox proportional hazards regression in SAS Studio (v3.81). Pearson correlation was performed to assess covariance in species relative abundance. A multivariable Cox proportional hazards model was then applied to assess the association between *S. aureus* detection and mortality risk. Confounders with a biologically plausible link to mortality or OP microbiome disruption were selected in consultation with clinician specialists. Variables included demographic characteristics (age, sex, days in residence), morbidity markers (overall comorbidity count, prior hospitalisation, dementia and medications indicated for psychosis, diabetes and pain [detailed in Appendix 1]) and OP microbiota modifiers (texture modified diet, lung disease, proton pump inhibitor dispensation). These variables were sourced from facility records, Pharmaceutical Benefits Scheme prescription data, Medicare Benefits Schedule (MBS) healthcare utilisation data, Aged Care Funding Instrument (ACFI) assessments [17] and Rx-Risk tool comorbidity data [18]. Hazard ratios (HR) and 95% confidence intervals (CI) were reported.

Categorical variables were compared between *S. aureus* carriers and non-carriers using Fisher’s Exact test. Non-parametric continuous variables were compared using the Mann–Whitney U test.

Factors associated with *S. aureus* carriage were analysed by multivariable logistic regression. Explanatory variables included those linked with oral microbiome modification or increased healthcare utilisation (Appendix 1). Odds ratios (OR) and 95% CIs are reported. A Type 1 error rate (α) of 0.05 was applied throughout. *P*-values were adjusted for multiple testing using Benjamini–Hochberg correction at a false discovery rate of 0.05.

## Results

OP swabs and healthcare utilisation data were obtained for 190 long-term aged care residents (Table 1). Of 322 bacterial species detected, initial univariate screening identified positive associations with mortality for *S. aureus* (*n* = 7; HR [95% CI]: 4.3 [1.5–12.2], *P* = .006, *q* = 0.59) and *Prevotella multiformis* (*n* = 3; HR [95% CI]: 5.5 [1.3–23], *P* = .02, *q* = 0.92). Subsequent analysis focused on *S. aureus* due to the strength of its association and its wider clinical significance. The absence of strong correlations between the relative abundance of *S. aureus* and other bacterial species (*r* < 0.42 for all) suggested the association with mortality was independent of other microbes.

**Table 1:** Cohort characteristics, including comparison by OP *Staphylococcus aureus* carriage status determined using qPCR

Variable	Total cohort (*n* = 190)	*Staphylococcus aureus* carriers (*n* = 13)	*Staphylococcus aureus* non-carriers (*n* = 177)	*P*-value
Age (years), median [IQR]	88.3 [81.3–92.6]	87.8 [81.4–94]	88.3 [81.3–92.6]	0.65
Sex (female), *n* (%)	135 (71.1)	9 (69.2)	126 (71.2)	1.00
12-month all-cause mortality, n (%)	35 (18.4)	9 (69.2)	26 (14.7)	<0.0001
Days since entry, median [IQR]	597.5 [259–1031]	558 [69–1370.5]	599 [260.5–1026]	0.80
Prior hospitalisation (12 months), *n* (%)	74 (38.9)	5 (38.5)	69 (40.0)	0.15
Prior respiratory infection (respiratory antibiotics, 12 months)	60 (31.6)	7 (53.8)	53 (29.9)	0.12
**Comorbidities, *n* (%)**				
Texture modified diet^a^	42 (22.1)	6 (46.2)	36 (20.3)	0.04
Depression	83 (43.7)	9 (69.2)	74 (41.8)	0.08
Psychosis	26 (13.7)	4 (30.8)	22 (12.4)	0.08
Diabetes	32 (16.8)	4 (30.8)	28 (15.8)	0.24
Pain	86 (45.3)	9 (69.2)	77 (43.5)	0.09
Lung disease	69 (36.3)	7 (53.8)	62 (35)	0.23
Dementia^a^	96 (50.5)	7 (53.8)	89 (50.3)	1.00
Cumulative number of comorbidities per participant, median [IQR]	5 [3–7]	7 [6–9.5]	5 [3–7]	0.02
**Medications, *n* (%)**				
Antibiotics (prior 90 days)	71 (37.4)	5 (38.5)	66 (37.3)	1.00
Proton pump inhibitors (prior 90 days)	87 (45.8)	7 (53.8)	80 (45.2)	0.58
Immunosuppressives (prior 12 months)	56 (29.5)	3 (23.1)	53 (29.9)	0.76

^a^Extracted from aged care funding instrument data.

Targeted qPCR confirmed initial detection of *S. aureus* in seven participants and identified carriage in a further six (total *n* = 13, 6.8%). Initial analysis identified *S. aureus* carriers as more likely to receive a texture modified diet due to swallowing impairment (46.2% carriers vs 20.3% non-carriers, *P* = .04), and exhibit a higher median number of comorbidities (median [IQR]: 7 [6–9.5] vs 5 [3–7], *P* = .02; Table 1). Thirty-five (18.4%) participants died during the 12-month follow-up, of whom nine were *S. aureus*-positive.

**Figure 1:**
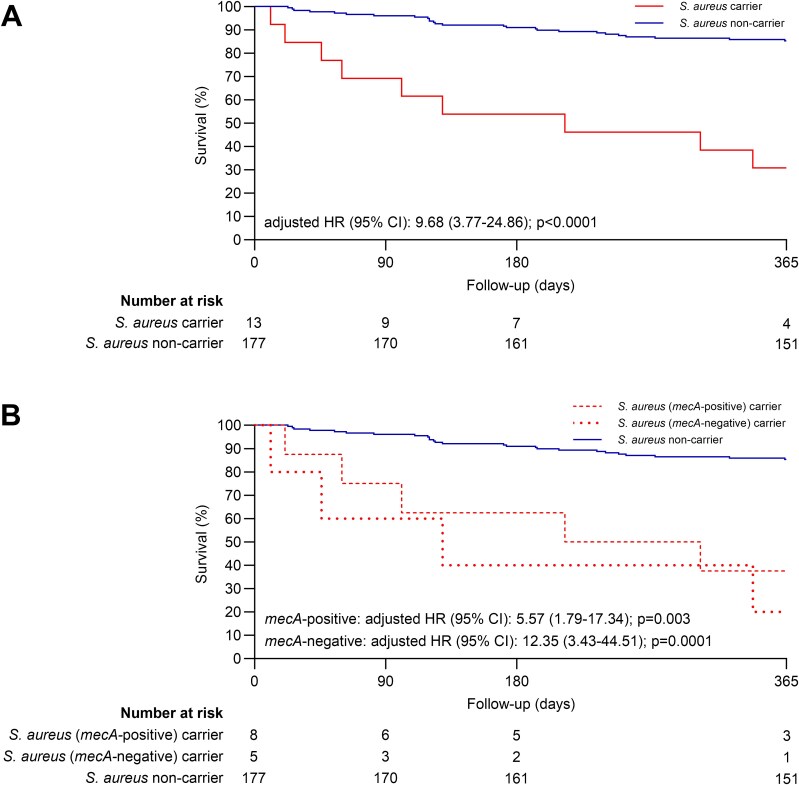
(A) Kaplan–Meier 12-month all-cause mortality estimates among OP *S. aureus* carriers and non-carriers. *Staphylococcus aureus* carriage was determined by detection of the *nuc* gene using qPCR. (B) Kaplan–Meier 12-month all-cause mortality estimates among *S. aureus* carriers that were also *mecA*-positive (dashes) and *mecA*-negative (dots) compared to non-carriers. Adjusted HR, 95% CI and *P*-values are reported for all.

Multivariable Cox regression revealed a strong association between qPCR-based *S. aureus* detection and 12-month mortality risk (adjusted HR [95% CI]: 9.7 [3.8–24.9], *P* < .0001; Figure 1A). This was independent of other assessed covariates and represented the strongest observed relationship in univariate analyses (Appendix 2). Carriage of methicillin-resistant *S. aureus* (MRSA) was assessed by *mecA* qPCR. Of the 13 *S. aureus*-positive participants, five carried the *mecA* gene. The association between *S. aureus* detection and mortality using the same approach remained significant for both *mecA* carriers (adjusted HR [95% CI]: 5.6 [1.8–17.3], *P* = .003) and non-carriers (adjusted HR [95% CI]: 12.4 [3.4–44.5], *P* = .0001; Figure 1B).

Multivariable logistic regression identified a significant, independent association between number of comorbidities and *S. aureus* detection (adjusted OR [95% CI]: 1.4 [1, 2], *P* = .04).

## Discussion

*Staphylococcus aureus* is a prevalent nosocomial pathogen often resistant to multiple antibiotics and capable of causing a wide range of infections [19]. Previous, culture-based, investigations of upper airway *S. aureus* carriage and mortality have reported an association with MRSA, but not MSSA. Mendy et al. (2016) reported analysis of 10,598 adults within the general population, in which MRSA colonisation predicted mortality prior to adjustment for socioeconomic status and comorbidities [20]. Within aged care populations, studies have consistently reported a higher mortality risk among MRSA carriers, but no relationship with MSSA [21–23]. These findings potentially reflect the omission of direct assessments of MSSA carriage.

Based on these prior studies, a presumption may exist that the significance of *S. aureus* carriage in the oropharynx relates to MRSA only. However, we report a robust positive association between OP *S. aureus* carriage and mortality in aged care residents that is independent of *mecA* status. This finding could be attributable to our utilisation of comprehensive and targeted molecular techniques.

While the basis for the relationship between *S. aureus* carriage and mortality is uncertain, we suggest two principal explanations. First, this relationship could reflect a direct contribution of *S. aureus* to health outcomes through an increased risk of potentially fatal infections, such as aspiration pneumonia or bacteraemia [24, 25]. This hypothesis could not be tested as access to reliable cause-of-death data was unavailable (a limitation of our study). However, our finding that this relationship was independent of methicillin resistance suggests such a direct contribution to mortality is unlikely.

Second, *S. aureus* colonisation of the oropharynx could represent a marker of other contributors to mortality risk. For example, the number of comorbidities was an independent predictor of *S. aureus* detection, suggesting that *S. aureus* could reflect a high disease burden, and, by extension, greater interaction with health services.

Should the relationship between OP *S. aureus* carriage and mortality be shown to principally reflect wider determinants of health, the question of clinical utility arises. Current approaches to assess disease burden involve the application of composite indices, such as the Charlson Comorbidity Index [26] and Elixhauser Score [27]. These validated indices predict mortality based on the presence and severity of comorbid conditions. It was notable that *S. aureus* carriage outperformed overall comorbidity count (a contributor to these indices) at a univariate level, and even after adjustment for multiple factors. While we do not suggest that OP *S. aureus* carriage represents an alternative to these composite indices, its inclusion might improve their performance.

Our study had limitations that should be considered. First, the prevalence of OP *S. aureus* in the study population was relatively low, requiring confirmation in a larger population. Second, assessment of healthcare interactions based on prescribed medications will fail to capture other aspects of service utilisation that could contribute to *S. aureus* acquisition, such as interactions with RACF nursing staff, outpatient clinics or access to primary health services. Third, the use of *mecA* detection as a basis for inferring MRSA carriage is imperfect, due to the potential carriage of this gene by *Staphylococcus epidermidis*. We attempted to mitigate the risk of misattribution by performing *mecA* qPCR only on samples that were *nuc*-positive. Fourth, the Rx-Risk comorbidity index assumes all prescriptions were taken as intended and may underrepresent conditions for which medication was not prescribed. Fifth, we were not able to assess host factors, such as subclinical immunological changes, chronic inflammation or unmeasured aspects of frailty, that may contribute to *S. aureus* carriage.

Further research is now required to substantiate our findings and investigate the basis of the relationship between OP *S. aureus* carriage and 12-month all-cause mortality. We suggest that these investigations focus on three issues: identification of factors that contribute to *S. aureus* carriage, the potential causal contribution of *S. aureus* to health outcomes and the relationship between carriage of *S. aureus* carriage and health outcomes other than mortality.

## Supplementary Material

aa-24-1268-File002_afaf042

## Research Data Transparency and Availability

Metagenomic sequence data for the GRACE study are accessible from the European Nucleotide Archive under the accession number PRJEB51408. Other de-identified datasets are available upon request. Access to metadata containing potentially identifying patient information requires an approved research ethics protocol and may require an approved material/data transfer agreement from the Southern Adelaide Clinical Human Research Ethics Committee.
