# Supplementary material for: Oropharyngeal Staphylococcus aureus is linked to higher mortality in long-term aged care residents
Source: Age Ageing. 2025 Mar 3;54(3):afaf042. doi: 10.1093/ageing/afaf042 (PMC12264333; doi:10.1093/ageing/afaf042)
Supplement: aa-24-1268-File002_afaf042 [file aa-24-1268-file002_afaf042.pdf]

Oropharyngeal *Staphylococcus aureus* is linked to higher mortality in long-term aged care residents

SUPPLEMENTARY MATERIAL

<u>Table of Contents</u>

Appendix 1: Supplementary methods..2

Appendix 2: Supplementary table 1..4

Appendix 1: Supplementary methods

Oropharyngeal swab DNA extraction

DNA from OP swabs was extracted using the ZymoBIOMICS miniprep kit (Zymo Research, CA, USA). Swabs were spun down at 3374 x g for 5 min to collect all biological material and the resultant solution was added to a bead-beating tube containing 750 µl of lysis buffer. Samples underwent bead-beating for 1 min 5 times at a speed of 6.5m/s in a FastPrep®-24 Homogenizer (MP Biomedicals, CA, USA) for a total of 5 min with 5 min rest in between each run. Samples were centrifuged at 10,000 x g for 2 min then 700 µl was added to the III-F filter in a clean tube and centrifuged at 8,000 x g for 1 min. Filtered solution was transferred to a clean tube and 2100 µl of DNA binding buffer was added. Samples were vortexed vigorously then 800 µl of solution was added to a IICR filter and centrifuged at 10,000 x g for 1 min. Flow through was discarded and this process was repeated until all solution had been passed through the filter. After transferring the filter to a new tube, 400 µl of the first wash buffer was added to the filter and centrifuged at 10,000 x g for 1 min. Flow through was discarded then 700 µl of a second wash buffer was added to the filter, centrifuged at 10,000 x g for 1 min and a final 200 µl of wash buffer was added to ensure all wash buffer had passed through. Filters were transferred to a new clean tube and 100 µl of dH2O at 60°C was added and incubated for 5 min. After centrifuging at 10,000 x g for 1 min, samples were added to a final spin column for purification and centrifuged at 16,000 x g for 3 min. DNA was stored at -80°C until further processing.

Metagenomic sequencing

Oropharyngeal swab DNA extracts underwent metagenomic library preparation using the Nextera XT DNA Library Prep Kit (Illumina, CA, USA) as per manufacturer's instructions, alongside blank and mock community controls. Libraries were normalised with Qubit assay, and pooled libraries were then sequenced on an Illumina Novaseq6000 platform with a 2 × 150 setup. Sequences were quality-filtered using Trimmomatic (v0.39) and reads that aligned to the NCBI human reference genome (release GRCh38) were removed using Bowtie (v2.3.5.1) [1, 2]. The resulting quality filtered, non-human reads had a median count of 47.8 million reads per sample (range: 33.6-56.7). Microbiome taxonomic composition data was determined using MetaPhlAn (v3.0) [3].

Covariate definitions

The multivariable Cox proportional hazards model adjusted for a variety of covariates selected based on biological plausibility and potential confounding effects. The adjustment for medication use was based on specific ATC codes corresponding to psychosis (N05AA01–N05AB02, N05AB06–N05AL07, N05AX07–N05AX13), diabetes (A10AA01–A10BX99), and pain (M01AB01–M01AH06; N02AA01–N02AX02, N02AX06, N02AX52, N02BE51). Medication usage based on ATC codes was derived from PBS prescription data.

Factors associated with *S. aureus* carriage were analysed using multivariable logistic regression. Explanatory variables included age, sex, days in residence, prior hospitalisation, comorbidity count, and a modified diet indicative of swallowing impairment. Respiratory infections in the 12 months prior to sample collection were included, as well as dispensing history for antibiotics, proton pump inhibitors (PPIs), anticholinergic medications (indicated for depression or psychosis), and immunosuppressive medications in the 12 months prior.

References

1. Bolger AM, Lohse M, Usadel B; Trimmomatic: a flexible trimmer for Illumina sequence data. *Bioinformatics* 2014;**30**(15):2114-20. doi: 10.1093/bioinformatics/btu170.
2. Langmead B, Salzberg SL; Fast gapped-read alignment with Bowtie 2. *Nat Methods* 2012;**9**(4):357-359. doi: 10.1038/nmeth.1923.
3. Beghini F, McIver LJ, Blanco-Míguez A, et al.; Integrating taxonomic, functional, and strain-level profiling of diverse microbial communities with bioBakery 3. *Elife* 2021;**10**. doi: 10.7554/eLife.65088.

Appendix 2: Supplementary table 1.

Staphylococcus aureus detection by qPCR and 12-month all-cause mortality risk in 190 aged care residents. A Cox proportion hazards regression model was used. The hazard ratio (HR) with 95% confidence intervals (CI) is reported with the associated *P* value. Univariate analyses were corrected for multiple testing at a false discovery rate of 0.05. All listed variables were included in the multivariable analysis.

	**Univariate analysis**			**Multivariable analysis**	
**Variable**	**HR (95% CI)**	***P* value**	**Adj *P* value**	**HR (95% CI)**	***P* value**
Staphylococcus aureus detection	7.22 (3.37-15.47)	<0.0001	<0.0001	9.68 (3.77-24.86)	<0.0001
Age	1.03 (0.98-1.08)	0.25	0.45	1.05 (0.99-1.10)	0.12
Sex	1.57 (0.79-3.12)	0.20	0.42	2.25 (1.07-4.76)	0.03
Days in residence	1.00 (1.00-1.00)	0.87	0.93	1.00 (1.00-1.00)	0.68
Prior Hospitalisation	1.05 (0.54-2.05)	0.89	0.93	0.61 (0.27-1.36)	0.22
Total comorbidity count	1.13 (1.01-1.26)	0.03	0.19	1.12 (0.95-1.33)	0.18
Dementia	1.36 (0.70-2.66)	0.37	0.58	1.49 (0.71-3.11)	0.29
Texture modified diet	1.02 (0.46-2.24)	0.96	0.93	1.21 (0.50-2.97)	0.67
Lung Disease	1.57 (0.81-3.06)	0.18	0.42	1.13 (0.54-2.36)	0.75
Pain	1.74 (0.89-3.39)	0.11	0.38	1.19 (0.53-2.64)	0.68
Psychosis	1.37 (0.57-3.29)	0.49	0.69	0.91 (0.34-2.40)	0.84
Diabetes	1.25 (0.54-2.85)	0.60	0.76	0.55 (0.21-1.46)	0.23
PPI use	1.70 (0.87-3.32)	0.12	0.38	1.61 (0.75-3.47)	0.23
